# Macular Rash, Thrombocytopenia, and Hyperbilirubinemia in a Preterm Infant

**DOI:** 10.1155/2019/4076740

**Published:** 2019-04-08

**Authors:** Nurin Chatur, Marina Castro, Kin Fan Young Tai

**Affiliations:** ^1^University of Toronto, Toronto, Ontario, Canada; ^2^Mount Sinai Hospital, Toronto, Ontario, Canada

## Abstract

Neonatal hyperthyroidism is usually caused by the passage of maternal thyroid receptor antibodies. This relatively rare condition has various manifestations including cholestasis, prematurity, and cardiomegaly. We present a case of a preterm infant with neonatal Graves' disease who presented with cholestasis, cardiomegaly, and a macularpapular rash that was thought to be suspicious for congenital infection. This case has been reported to illustrate lessons learnt for early identification of a neonate with Graves' disease in order to expedite treatment.

## 1. Introduction

Neonatal Graves' disease (GD) is a rare disease found in infants affected by transplacental passage of maternal thyroid-stimulating antibodies, which binds to the thyroid-stimulating hormone receptor (TSHR) and leads to increased production of thyroid hormone [1]. Fetal thyroid development is established by 7 weeks of gestation, and thyroid hormone synthesis begins between 10–12 weeks of gestation. The thyroid is considered functionally mature by 25 weeks, and thus, transfer of maternal TRAb to the fetus can cause in utero or postnatal hyperthyroidism [2]. Maternal Graves' disease is present in approximately 0.2% of pregnancies, and neonatal Graves' is seen in around 1% of those cases or in 1/50000 neonates [1, 2].

Various different signs and symptoms have been associated with the diagnosis. Clinical findings such as hyperthermia, irritability, feeding intolerance, heart failure, tachycardia, and hepatosplenomegaly are among the constellation of symptoms that are associated with Graves' disease [3]. Laboratory findings include low or fully suppressed thyroid-stimulating hormone levels (TSH), elevated thyroid-stimulating receptor antibodies (TSAbs), elevated free T3 and T4, and transaminitis and thrombocytopenia [1]. Because often the clinical signs and symptoms are nonspecific and can also be seen in sepsis and congenital viral infection, neonatal Graves' can be misdiagnosed [4]. Misdiagnosis can lead to preventable morbidity and mortality with rates of up to 20% mortality seen in neonatal Graves' [5].

## 2. Case Description

**Figure 1:**
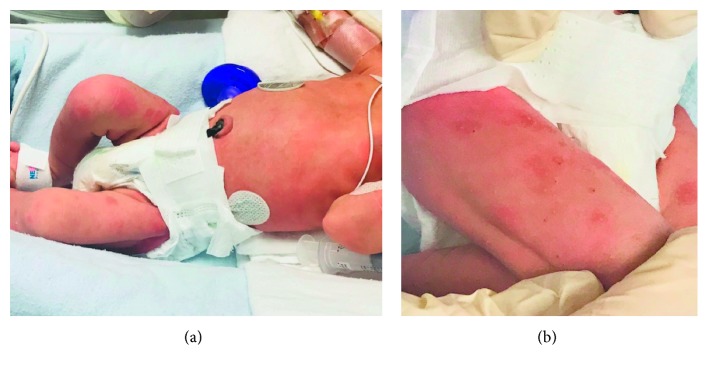
Photographs of the neonate's diffuse macular rash.

A 32-week gestational-age male infant was born to a 38-year-old G4P1 mother with premature prolonged rupture of membranes (PPROM) of 2 days duration. On the third trimester scan, the fetus was found to have cardiomegaly and splenomegaly. The neonate was delivered by caesarean section due to persistent fetal tachycardia and presence of meconium-stained amniotic fluid. Pregnancy history was otherwise unremarkable with protective maternal serologies including negative HIV testing, negative syphilis testing, immunity to hepatitis B, and immunity to rubella. Maternal history was significant for hypothyroidism, adequately treated on levothyroxine. At birth, he required positive pressure ventilation. Once stabilized, he was noted to have diffuse erythematous macules 2-3 mm diameter and well-defined borders over back, trunk, and extremities as shown in Figure 1. A more detailed physical exam revealed a tachycardiac, nondysmorphic neonate, of birth weight 1760 g (50^th^ percentile), head circumference 30 cm (66^th^ percentile), and length 44 cm (78^th^ percentile). Cardiovascular exam was normal aside from tachycardia. Respiratory exam revealed increased work of breathing that improved on CPAP. Abdominal examination showed the liver was 2 cm below right costal margin and the spleen 3 cm below left costal margin, otherwise was soft and not tender. Rest of exam was otherwise unremarkable. His initial CBC showed a Hb of 186 g/L, WBC of 32.6 × 10^9^/L, and platelets of 47 × 10^9^/L. His initial liver enzymes were as follows: GGT 600 unit/L, ALP 209 unit/L, AST 82 unit/L, ALT 24 unit/L, INR 1.7, PT 18.3 secs, and APTT 30.2 secs. A 12-hour bilirubin level revealed total 219 *μ*mol/L with direct 83 *μ*mol/L and indirect 136 *μ*mol/L.

The patient continued to be persistently tachycardiac and hypertensive. A septic workup was done, and he was treated empirically with antibiotics. He was treated with phototherapy for the indirect component of his hyperbilirubinemia and received platelet transfusions for the thrombocytopenia. The presumed diagnosis was congenital infection. TORCH studies were sent and were negative. Due to elevated liver enzymes with cardiomegaly and hepatosplenomegaly, Genetics and Gastroenterology were consulted. A metabolic workup was done. Significant results were as follows: T4 greater than 100 pmol/L, TSH less than 0.01 mIU/L, and free T3 of 27 pmol/L. Thyroid-stimulating receptor antibodies were found to be elevated at 12.1 IU/L (normal <1.0 IU/L). His neck ultrasound showed a homogenously enlarged thyroid. Endocrinology was consulted and diagnosed neonatal Graves' disease. On further history, his mother revealed that she was hypothyroid secondary to Graves' disease treated with radioablative therapy many years prior to immigrating to Canada. Methimazole and propranolol were started. His tachycardia and hypertension improved, and his free T4 level decreased. Additionally, his rash resolved. An echocardiogram ruled out pulmonary hypertension and showed small PDA and fenestrated atrium. He required two platelet transfusions due to thrombocytopenia in the first week of his admission. His platelet levels normalized prior to discharge. During the course of treatment, he developed worsening transaminitis secondary to the methimazole. Thus, he was weaned from methimazole and treated with iodine. His bilirubin levels stabilized with total bilirubin 167 *μ*mol/L and direct bilirubin 109 *μ*mol/L. His transaminitis improved with GGT 292 unit/L, AST 196 unit/L, and ALT 119 unit/L. His thyroid function tests normalized at the time of discharge with a T4 level of 12 pmol/L, TSH less than 0.01 mIU/L, and T3 of 2.9 pmol/L. Iodine and propranolol were discontinued prior to discharge home.

## 3. Discussion

Neonatal Graves' disease is usually self-limited. However, it can have serious effects on the patient and can manifest itself in a variety of ways. Remission usually occurs between 20 and 48 weeks of life [6]. Previously observed manifestations of neonatal GD include prematurity, growth restriction, hypertension, tachycardia, thrombocytopenia, hepatitis, cholestatic jaundice, goiter, and pulmonary hypertension [5]. The timing of the symptoms is variable with the majority of patients diagnosed within the first two weeks of life. Often, fetal hyperthyroidism can be seen in the third trimester including tachycardia, heart failure, premature labour, and intrauterine growth restriction. Overt neonatal hyperthyroidism can also be noted at birth, but there can be a delay in the manifestations of this condition if there is active maternal treatment ongoing during the pregnancy [3].

Remission usually occurs between 20 and 48 weeks of life [6]. Neonatal Graves' has also been reported in a neonate with a mother who had autoimmune hypothyroidism with transplacental passing of TSAbs [7].

Hyperthyroidism is not typically thought to be associated with cholestasis; however, it has been reported in both the pediatric and adult population [8–10]. There are several explanations for liver dysfunction in hyperthyroidism. Animal studies have shown that T3 can cause hepatic apoptosis [9]. T3 is involved in bilirubin mechanism, and increased levels are thought to cause accumulation of bilirubin precursors. Furthermore, as discussed earlier, hyperthyroidism can lead to increased cardiac output and heart failure which can also contribute to abnormalities in liver function [8].

In this report, we describe an unusual case of neonatal Graves' disease presenting with prematurity, cholestasis, and macular rash. The rash with cholestasis combined with the abnormalities on prenatal ultrasound and unknown history of maternal Graves' led to an initial infectious investigation. However, the rash has also been linked to other cases of neonatal Graves' and is not a finding isolated to congenital infection [5, 6]. The findings of the patient having fetal cardiomegaly, splenomegaly, and tachycardia are nonspecific and, in retrospect, were indicators of the diagnosis of neonatal Graves'.

In the situation where there is a known maternal history of Graves' disease, TRAb should be taken from the cord blood. If not available, this should be drawn from the neonate. If negative, the neonate's free T4 and TSH test should be done between days of life 3–5 and repeated again between days of life 10–14 [1]. Treatment in the acute period generally involves methimazole. Betablockers can be added for sympathetic hyperactivity as seen in our case described above. If patients are refractive, you can consider potassium iodide therapy in conjunction with methimazole or alone. In the long term, central or primary hypothyroidism can occur in newborns afflicted with neonatal Graves' disease [1]. As neonatal Graves' can be associated with cholestasis, liver enzymes should be followed, especially when using methimazole. A rare known side effect of methimazole is hepatic dysfunction, often of a cholestatic picture [11].

As illustrated by our case, if a clinician notes that the neonate has a macular rash with cholestasis and tachycardia in the context of maternal hypothyroidism, he/she should broaden their differential diagnosis to include neonatal Graves' disease. With a maternal history of Graves' disease, maternal TRAbs levels can be measured during pregnancy as maternal Graves' disease may influence management in the perinatal period [2]. The diagnosis in this case was difficult to make due to the mother's remote history of Graves' disease that had been treated with ablative therapy. Her subsequent hypothyroidism was also adequately managed with levothyroxine. However, thyroid receptor antibodies may still cross the placenta and cause Graves' in a fetus or neonate even though the mother was euthyroid and asymptomatic.

This case illustrates the importance of a thorough history taking during pregnancy and at delivery as well as general awareness of the varied presentation of neonatal Graves'.

## References

[1] Polak M., Legac I., Vuillard E., Guibourdenche J., Castanet M., Luton D. (2006). Congenital hyperthyroidism: the fetus as a patient. *Hormone Research in Paediatrics*.

[2] Besançon A., Beltrand J., Le Gac I., Luton D., Polak M. (2014). Management of neonates born to women with Graves’ disease: a cohort study. *European Journal of Endocrinology*.

[3] van der Kaay D. C. M., Wasserman J. D., Palmert M. R. (2016). Management of neonates born to mothers with Graves disease. *Pediatrics*.

[4] Caroll D., Kamath P., Stewart L. (2005). Congenital viral infection?. *The Lancet*.

[5] Oglivy-Stuart A. L. (2002). Neonatal thyroid disorders. *Archives of Disease in Childhood—Fetal and Neonatal Edition*.

[6] Zimmerman D. (1999). Fetal and neonatal hyperthyroidism. *Thyroid*.

[7] Joshi K., Zacharin M. (2018). Hyperthyroidism in an infant of a mother with autoimmune hypothyroidism with positive TSH receptor antibodies. *Journal of Pediatric Endocrinology and Metabolism*.

[8] Fatima S., Puri R., Patnaik S., Mora J. (2017). When a toxic thyroid makes the liver toxic: a case of thyroid storm complicated by acute liver failure. *AACE Clinical Case Reports*.

[9] Khadora M. M., Dubayee M. A. (2014). Neonatal Graves’ disease with unusual metabolic association from presentation to resolution. *BMJ Case Reports*.

[10] Loomba-Albrecht L. A., Bremer A. A., Wong A., Philipps A. F. (2012). Neonatal cholestasis caused by hyperthyroidism. *Journal of Pediatric Gastroenterology and Nutrition*.

[11] Livadas S., Xyrafis X., Economou F. (2010). Liver failure due to antithyroid drugs: report of a case and literature review. *Endocrine*.

